# A Soft Label Method for Medical Image Segmentation with Multirater Annotations

**DOI:** 10.1155/2023/1883597

**Published:** 2023-02-18

**Authors:** Jichang Zhang, Yuanjie Zheng, Yunfeng Shi

**Affiliations:** School of Information Science & Engineering, Shandong Normal University, No. 1 Daxue Road, Changqing District, Jinan 250358, China

## Abstract

In medical image analysis, collecting multiple annotations from different clinical raters is a typical practice to mitigate possible diagnostic errors. For such multirater labels' learning problems, in addition to majority voting, it is a common practice to use soft labels in the form of full-probability distributions obtained by averaging raters as ground truth to train the model, which benefits from uncertainty contained in soft labels. However, the potential information contained in soft labels is rarely studied, which may be the key to improving the performance of medical image segmentation with multirater annotations. In this work, we aim to improve soft label methods by leveraging interpretable information from multiraters. Considering that mis-segmentation occurs in areas with weak supervision of annotations and high difficulty of images, we propose to reduce the reliance on local uncertain soft labels and increase the focus on image features. Therefore, we introduce local self-ensembling learning with consistency regularization, forcing the model to concentrate more on features rather than annotations, especially in regions with high uncertainty measured by the pixelwise interclass variance. Furthermore, we utilize a label smoothing technique to flatten each rater's annotation, alleviating overconfidence of structural edges in annotations. Without introducing additional parameters, our method improves the accuracy of the soft label baseline by 4.2% and 2.7% on a synthetic dataset and a fundus dataset, respectively. In addition, quantitative comparisons show that our method consistently outperforms existing multirater strategies as well as state-of-the-art methods. This work provides a simple yet effective solution for the widespread multirater label segmentation problems in clinical diagnosis.

## 1. Introduction

Recently, deep learning techniques have made impressive progress on image segmentation tasks and have become a popular choice in the computer vision community [1]. Typically, supervised learning in deep learning is based on the assumption that there is a ground truth (GT). However, the truth is a lie; that is, there is often a lack of human consensus on the category of an object [2–4]. Especially, in medical image segmentation, which is based on knowledge and experience, disagreements between raters are fairly common [5, 6]. Inter-rater variability, as frequently reported by relevant research in the clinical field, usually leads to difficulties in segmenting areas of high uncertainty [7, 8].

To mitigate this inter-rater variability, the most basic yet common approach is the majority voting approach, in which opinions agreed by a majority of raters are taken as true. However, the majority voting approach essentially discards the rich information contained in the multirater labels through one-hot operation (e.g., the probability distribution [0.6, 0.3, and 0.1] is transformed into a hard label [1, 0, and 0]). To combat this issue, soft-label methods that average rater annotations have been intensively investigated [9, 10]. Furthermore, Islam and Glocker [11] introduced a label smoothing method that incorporates fuzzy information about edges into multirater soft labels, called spatially varied label smoothing (SVLS).

**Figure 1:**
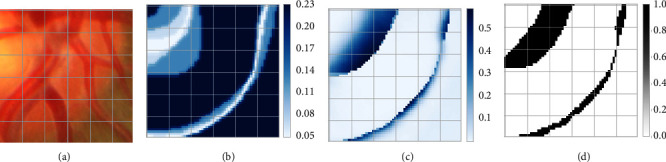
(a) Local visualization of an exemplary fundus image; (b) interclass variance map of annotations from six raters for OC and OD segmentation; (c) corresponding loss map of prediction; (d) corresponding error rates' map of prediction.

**Figure 2:**
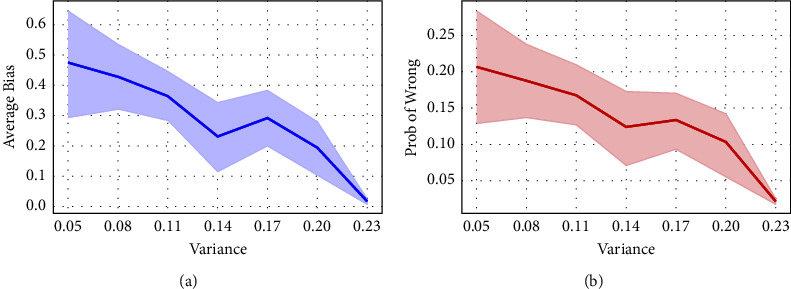
(a) Line graph of pixelwise interclass variances versus pixelwise loss; (b) line graph of pixelwise interclass variances versus pixelwise probability of misprediction. The abovementioned statistics are averaged on the validation set.

However, when we applied the soft labels method to the multirater optic cup (OC) and optic disc (OD) segmentation of the fundus image task, finding that the areas where segmentation errors occur coincides with the highly divergent areas to some extent, see Figure 1. As demonstrated in Figure 2, the pixelwise loss and error rates of predictions are statistically positively correlated with the interclass variance which indicates the divergence between raters. We tentatively attempt to explain this phenomenon as follows:

- An explanation is that the divergence area is highly uncertain, and the higher the uncertainty of the label, the lower the penalty imposed on the predicted distribution [12]. Highly uncertain annotations make it difficult to impose strong and precise constraints on the model, which is similar to weakly supervised learning that lacks accurate annotations [13]. The dependence on annotations in weakly supervised learning is weakened and replaced by a focus on features [14].
- Furthermore, we provide an intuitive interpretation that is more consistent with the multirater labels segmentation task: uncertainty reflects pixelwise image difficulty, where areas with high difficulty are more challenging for the model to accurately segment. Image difficulty, which is related to the visual characteristics of the images, such as image quality and occlusion of the area of the lesions, is one of the causal factors of inter-rater variability [15]. As demonstrated in Figure 1(a), the blood vessels occluding the edge region of the OD not only make ophthalmologists' judgment difficult but also hinder the accurate prediction of the deep neural network. In contrast to existing methods [16, 17] that treat difficulty as image level, we innovatively consider difficulty to be pixelwise for segmentation tasks.

In conclusion, the regions with high inter-rater variability have more difficult features but only weaker supervision, which could be a cause of mis-segmentation. In this work, we aim to improve the performance level of the soft label approach on multirater labels' segmentation task based on the previously mentioned explanations. A way to get the best of both sides is to increase the focus on image features while reducing the reliance on highly uncertain annotations. Consequently, we propose a supervised segmentation network that is constrained by consistency regularization. Specifically, consistency regularization exploits the augmentation invariance of images to optimize the feature space while avoiding relying simply on labels and compensating for the disadvantage of unreliable local annotations. The uncertainty as the prior knowledge is formulated as the soft labels' interclass variance, which drives the proposed model's local difference training. In addition, the SVLS approach, which incorporates edge fuzziness into soft labels, is used to soften average expert labels.

Experiments are performed on a synthetic dataset with great disagreement as well as a real-world dataset. In these experiments, our method consistently outperforms existing multirater strategies and state-of-the-art (SOTA) methods. To verify the generalization of the proposed method, we additionally conduct generalization experiments on two other types of datasets.

In summary, the main contributions of this study are as follows:

1. To embed consistency/inconsistency of multirater into the model, the soft labels obtained by averaging softened annotations of raters are used as GT.
2. We provide thinking that disagreement among multiple raters, i.e., uncertainty, can be quantified from soft labels and used as prior knowledge to reflect the pixel-level difficulty of an image.
3. We propose to use consistency regularization to improve the model's attention to features and reduce dependence on GT, especially in regions of high uncertainty. Without introducing additional parameters, the accuracy of our method is improved over that of other methods on synthetic and real-world datasets.

## 2. Related Works

The problem of multirater labels' segmentation caused by inter-rater variability has started to pique the interest of researchers. There is a study showing that the observed labels depend on three causal factors: the true label, the expertise of the rater, and the image difficulty [16]. For the method of obtaining the true label, it is a common practice to use majority voting [18] and STAPLE [19] or other label fusion strategies to obtain the ground-truth labels [11, 20] so that they can be adapted to the general segmentation model. However, simple label fusion methods neither do take advantage of any image features nor do they carry the inter-rater variability through to the model. Recently, several efforts have started to explore the expertise of the rater using label sampling strategies [21] or rater modeling strategies [22]. For instance, Zhang et al. [23] proposed to use confusion matrices to the model preference of annotators, obtaining segmentation prediction with the least noise by optimizing two coupled convolutional neural networks (CNNs). Yu et al. [15] proposed a multibranch model for the multirater glaucoma classification task, encouraging the specificity branch and the sensitivity branch to generate consistent/opposing predictions for consensus/disagreement samples. Ji et al. [24] proposed MRNet, which embeds the expertise of individual annotators into the model to generate calibrated predictions under different expertise levels for medical image segmentation.

However, there still lacks effective research on the image difficulty represented by image features in the multirater label segmentation task. Furthermore, we consider that multirater labels' segmentation is weakly supervised learning with inaccurate labels, which has not been explored before. Although our approach is uncertainty-driven, unlike works, such as Monte Carlo dropout [25] and ensembles [26, 27], that evaluate uncertainty and produce multiple segmentation hypotheses, our work aims to learn a deterministic single-output deep model.

## 3. Methodology

**Figure 3:**
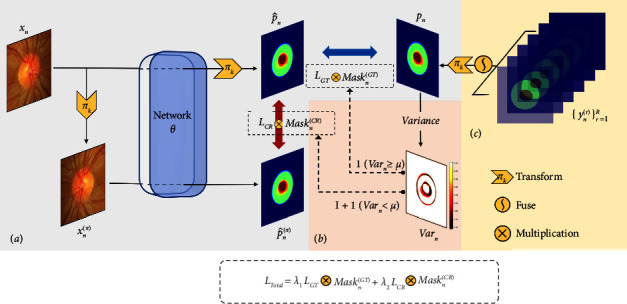
The architecture of our model consists of three parts: (a) segmentation network part; (b) asymmetrical regularization part; (c) multirater labels' fusion part.

The main architecture of our model is illustrated in Figure 3, which is composed of three main parts: (a) segmentation network with consistency regularization for conveying more information about the input; (b) asymmetrical regularization part for generating uncertainty mask to realize local self-ensembling in supervised learning; (c) multirater labels fusion part for obtaining a soft label for each input as the supervised target containing uncertainty. In the test and application phase, just the trained network is required to predict the segmentation of the input image.

### 3.1. Problem Definition

In this article, we consider the problem of learning a segmentation model from labels annotated by multiple human raters. Given the images {*X*^*W*×*H*×*L*^=*x*_*n*_}_*n*=1_^*N*^ and the corresponding one-hot labels {*Y*^*W*×*H*×*C*^=*y*_*n*_^(*r*)^}_*n*=1,⋯,*N*_^*r*=1,⋯,*R*^ (*W*, *H*, *L*, *C* denote the width, height, channels, and classes), where *N* is the number of samples and *R* is the number of raters, each image is independently annotated by raters based on their personal experiences. The objective of the multirater label segmentation task is to learn the projection function *F*(·), mapping the input image *x*_*n*_ to the estimated prediction ${\hat{y}}_{n}$ which is one-hot form encoded by the full probability distribution ${\hat{p}}_{n}$. In our article, ${\hat{p}}_{n}$ is encouraged to be as similar as *p*_*n*_, which is the soft label fused by *Y*_*n*_.

### 3.2. Soft Labels

Recently, increasing studies have proposed training a model using soft labels for accounting for the high uncertainty in lesion or structure borders' delineation [11, 28–31]. Averaging multirater labels is an intuitive way to obtain soft labels in multirater annotation tasks as follows:

(1) $$\begin{array}{c} p_{n}=\frac{1}{R}\overset{R}{\underset{r=1}{\sum }}y_{n}^{\left(r\right)}. \end{array}$$

Although the average strategy incorporates uncertainty from inter-rater variability into soft labels, it indulges the overconfidence of each rater. Therefore, we soften each hard label by SVLS and then average them to obtain *p*^*n*^ which contains spatial and inter-rater uncertainty as follows:

(2) $$\begin{array}{c} p_{n}^{\left(i,j\right)}=\frac{1}{R}\overset{R}{\underset{r=1}{\sum }}\frac{1}{\sum w}\overset{3}{\underset{a=1}{\sum }}\overset{3}{\underset{b=1}{\sum }}y_{n}^{r\left(i-a,j-b\right)}w^{\left(a,b\right)}\text{,} \end{array}$$

where (*i*, *j*) is the position of pixel and *w* is a weight matrix which is obtained by $1/\sqrt{2\pi \sigma^{2}}e^{-{\left|\overset{⟶}{x}\right|}^{2}/2\sigma^{2}}$ with *σ* = 1. SVLS determines the probability of the target pixel based on its neighboring pixels, achieved by a Gaussian-like weight matrix that is applied across the one-hot encoded rater labels *y*^(*r*)^ to obtain a soft probability distribution.

The transmission of uncertainty information into the model is inseparable from the appropriate loss function. There is the performance of several common loss functions in Section 4.4, including soft cross-entropy loss, soft dice loss, and soft focal loss. By comparison, the soft cross-entropy loss is selected as the optimization objective, encouraging the probability distribution of prediction ${\hat{p}}_{n}$ to be identical to that of the soft label as follows:

(3) LGT=−∑n=1Npnlogp^n.

### 3.3. Label Uncertainty Measure

It is equally crucial to model uncertainty at the pixel-level as to improve the model's performance, particularly in medical scenarios [25]. Unlike work that uses stochastic networks [32, 33] to model uncertainty, we improve multirater models using uncertainty as a source of prior knowledge. Specifically, we consider the pixelwise interclass variance {Var_*n*_^*W*×*H*^}_*n*=1,⋯,*N*_ that reflects the uncertainty caused by inter-rater variability and spatial variation. It is inversely proportional to entropy, meaning that the lower the variance, the greater the entropy and, hence, the greater the uncertainty. The appropriate uncertainty can enhance the generalization and calibration of the model. However, the high uncertainty would be detrimental to the model as noise. In the position of the (*i*, *j*)^*th*^ pixel, the variance between classes Var(*p*_*n*_^(*i*, *j*)^) can be formulated by the following:

(4) $$\begin{array}{c} Var\left(p_{n}^{\left(i,j\right)}\right)=\frac{1}{C}\overset{C-1}{\underset{c=0}{\sum }}{\left(p_{n}^{\left(i,j\right)}\left(c\right)-\frac{1}{C}\right)}^{2}\text{,} \end{array}$$

where ∑_*c*=0_^*C*−1^*p*_*n*_^(*i*, *j*)^(*c*)=1. We propose to use uncertainty as a threshold to assign different optimization objectives to different areas of the image. Specifically, the labels of areas with high uncertainty are no longer decisive but are replaced with constraints on the feature space, which will be clarified in the next section. In areas with high rater agreement, soft labels and feature constraints work together to optimize the model. For convenience, we refer to this uncertainty-driven local differential optimization as asymmetrical regularization. The threshold comes into play in the form of a mask of 0-1, acting directly on the loss function. Mask is differentiated into mas*k*^(*GT*)^ and mas*k*^(*CR*)^ based on the threshold, which correspond to areas of low and high uncertainty, respectively, as follows:

(5) $$\begin{array}{c} mask_{n}^{\left(GT\right)}=1\left({Var}_{n}\geq \mu \right)\text{,}\\ mask_{n}^{\left(CR\right)}=I+1\left({Var}_{n}<\mu \right)\text{,} \end{array}$$

where *𝟙* is the indicator function and **I** is the identity matrix with the same shape as Var_*n*_.

### 3.4. Consistency Regularization

To improve attention to features and optimize feature space, we propose using consistency regularization as an extra constraint on the model, which has been utilized in semisupervised learning [34] and unsupervised learning [35]. Consistency regularization is a type of self-ensemble learning because it only relies on the images themselves to learn. Inspired by Li et al. [34], we apply rotation consistency to this work. Specifically, there is a problem in the segmentation task using CNN: when the inputs of CNN are rotated, the corresponding network predictions would not be rotated in the same way [36] as follows:

(6) $$\begin{array}{c} \theta \left(\pi_{k}x_{n}\right)\neq \pi_{k}\theta \left(x_{n}\right)\text{,} \end{array}$$

where *π*_*k*_ is a rotation to the image (i.e., horizontal, vertical, or mixed flip) and *θ* is the parameters of the network. The feature space is automatically optimized when the model is encouraged to make the same judgments about elements before and after rotation. In this article, we use the soft cross-entropy loss function as the optimization target of rotation consistency regularization term:

(7) $$\begin{array}{c} L_{CR}=-\overset{N}{\underset{n=1}{\sum }}\theta \left(\pi_{k}x_{n}\right)\log\;\left(\pi_{k}\theta \left(x_{n}\right)\right)\text{.} \end{array}$$

When formulas (3) and (7) are combined, the total loss function is as follows:

(8) $$\begin{array}{c} L_{total}=-\lambda_{1}\overset{N}{\underset{n=1}{\sum }}p_{n}\log\left({\hat{p}}_{n}\right)-\lambda_{2}\overset{N}{\underset{n=1}{\sum }}\theta \left(\pi_{k}x_{n}\right)\log\;\left(\pi_{k}{\hat{p}}_{n}\right)\text{,} \end{array}$$

where *λ*_1_ + *λ*_2_ = 1. By minimizing the loss function, the network is urged to focus more on the image content than on the regression of GT alone [37]. So far, image features can be fully expressed through self-ensembling. In regions of divergence where uncertainty is high, supervision of labels is entirely replaced by unsupervised self-ensembling. Without introducing extra parameters and structures, asymmetrical regularization is achieved by covering the soft label with an uncertainty-based mask (formulas (5) and (7)). Finally, updated formula (8) is shown as follows:

(9) $$\begin{array}{c} L_{total}=-\lambda_{1}mask_{n}^{\left(GT\right)}\overset{N}{\underset{n=1}{\sum }}p_{n}\log\left({\hat{p}}_{n}\right)+-\lambda_{2}mask_{n}^{\left(CR\right)}\overset{N}{\underset{n=1}{\sum }}\theta \left(\pi_{k}x_{n}\right)\log\;\left(\pi_{k}{\hat{p}}_{n}\right)\text{.} \end{array}$$

## 4. Experiments

In this section, we introduce the experimental dataset, implementation details, and evaluation metrics. In order to explore the best performance under different combinations of the loss and uncertainty threshold value, we conduct quantitative experiments with different setups on the MNIST and the RIGA validation set in Section 4.4. For comparison with other methods, the common label fusion approach and other SOTA approaches for multirater labels segmentation are used as the benchmark. The results are listed in Section 4.5, showing that our method can exploit the uncertainty of multirater annotations to improve segmentation performance. Additionally, ablation experiments are conducted to evaluate the efficacy of each component of our method.

### 4.1. Datasets

- MNIST is a handwritten digits dataset with 60,000 training and 10,000 test examples. All images are 28 × 28 grayscale versions of the handwritten numbers 0–9. Zhang et al. [23] synthesized a dedicated dataset of multirater annotation tasks based on MNIST, which simulates raters with different biases to obtain multiple labels by using Morpho-MNIST software [38]. Specifically, the first rater provides good segmentation with approximate GT, the second rater tends to oversegment, the third rater tends to undersegmentation, the fourth rater is prone to the combination of small fractures and oversegmentation, and the fifth rater always annotates everything as the background. We train a model using all five raters' annotations and finally test the model performance on GT.
- RIGA is a publicly available dataset for joint OC and OD segmentation from the University of Michigan [39]. It includes a total of 750 color fundus images from three subsets: 460 images from MESSIDOR, 195 images from BinRushed, and 95 images from Magrabia. Each fundus image has six OC and OD annotations carried out by six ophthalmologists. We select BinRushed and MESSIDOR as the training set, and Magrabia is selected as the test set, where all images are resized to 256 × 256. In accordance with the experimental design of MRNet [24] and other methods [11, 23], the majority voting of six raters for each test image is used as the silver standard to evaluate the prediction.
- QUBIQ-Kidney and Prostate are subdatasets of Quantification of Uncertainties in Biomedical Image Quantification Challenge (QUBIQ) [40], which are specifically designed to evaluate inter-rater variability. The QUBIQ-Kidney images are 2D CT slices (20 cases for training and 4 cases for testing) in which the kidneys are manually annotated by three raters. The QUBIQ-Prostate images are 2D MRI slices (48 cases for training and 7 cases for testing) in which the prostate is manually annotated by six raters. To match the task objective of the QUBIQ challenge, GT and prediction are binarized at five probability levels (0.1, 0.3, 0.5, 0.7, and 0.9), and evaluation scores for all thresholds will be averaged.

### 4.2. Implementation Details

For a fair comparison, we employ the same network architecture as the baseline approach. Specifically, for the MNIST experiment, we use the U-Net architecture without pretraining as [23].Moreover, for the RIGA experiment, the main framework utilizes the U-Net architecture with ResNet34 as the backbone. Parameters of the U-Net encoder are initialized with the pretrained model on ImageNet [41]. The abovementioned network is implemented with the PyTorch platform and trained/tested on a Tesla V100 GPU with 32 GB of memory. The proposed network is trained end-to-end using the Adam optimizer [42], and it takes about 4 hours to train our model with a mini-batch size of 4 for 60 epochs. The learning rate is set to 1 × 10^−4^.

### 4.3. Evaluation Metrics

Various evaluation metrics, including the Dice similarity coefficient (DSC) and mean intersection over union (mIoU), were utilized to evaluate the performance of the proposed method for segmenting OC and OD relative to GT. These performance metrics are defined as follows:

(10) $$\begin{array}{c} DSC\left(D\right)=\frac{2\times TP}{2\times TP+FP+FN}\text{,}\\ mIoU\left(I\right)=\frac{TP}{FP+FN+TP}\text{,} \end{array}$$

where TP, FP, FN, and TN represent true positives, false positives, false negatives, and true negatives, respectively, in the evaluation confusion matrix. Note that a model with higher metric values can predict more precise segmentation masks. All experimental results are reported as the average of the ten experiments conducted on the test set.

### 4.4. Performance of Our Methods

**Figure 4:**
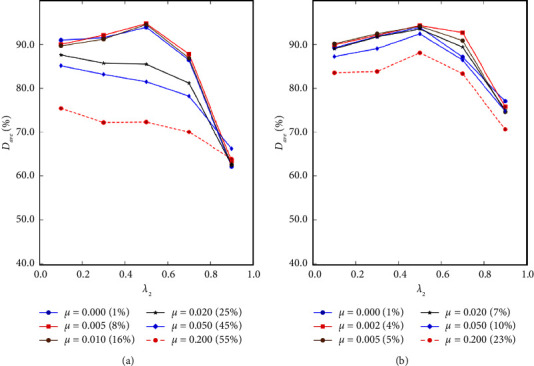
Segmentation accuracy under different *μ* and *λ*_2_ on the (a) MNIST and (b) RIGA.

Here, we provide a quantitative comparison among different loss functions including soft cross-entropy loss (CE), soft dice loss (DL), and soft focal loss [43] (FL).  Table 1 displays the top five combinations of loss functions with the highest *𝒟* under the corresponding optimal hyperparameter settings, including the uncertainty threshold *μ* and the unsupervised loss weight *λ*_2_, where experiments are performed on the MNIST validation set. The proposed method exhibits the best performance when both the supervision loss and the consistency regularization loss are CE. In Figure 4, we further present the comparison of model accuracy at different *μ* and *λ*_2_ settings under this loss function combination on the MNIST and RIGA validation set. Moreover, the average unsupervised proportion corresponding to different *μ* in the two datasets is performed in Figure 4. By comparison, the optimal (*μ*, *λ*_2_) combinations on the MNIST and RIGA datasets are (0.5, 0.005) and (0.5, 0.002), respectively, with corresponding unsupervised proportions of 8% and 4%.

### 4.5. Comparisons with Other Methods

To demonstrate the advantage of the proposed method, we compare our method to the SOTA methods on the MNIST and RIGA datasets. We use the publicly released code with default parameters to retrain the SOTA methods with the same training/test set as ours for a fair comparison.

Table 2 quantitatively compares our framework to three hard label methods, five soft label methods, and other SOTA multirater labels' segmentation methods, including (a) Mode-UNet: UNet trained using a single label randomly selected; (b) MV-UNet: UNet trained using one-hot labels obtained by majority voting; (c) STAPLE-UNet: UNet trained using one-hot labels obtained by STAPLE [19]; (d) Average-UNet: UNet trained using soft labels obtained by average raters [31]; (e) GLS-UNet: UNet trained using soft labels smoothed by general label smoothing [44]; (f) Sharpen-UNet: UNet trained using soft labels smoothed by label sharpen [45] under temperature (*T*) = 0.5 or 1.5; (g) Mixup-UNet: UNet trained using soft labels smoothed by Mixup; (h) SVLS-UNet: UNet trained using soft labels smoothed by SVLS [11]; (i) LNL [23]; (j) MRNet [24] on the MNIST and RIGA test sets.

**Figure 5:**
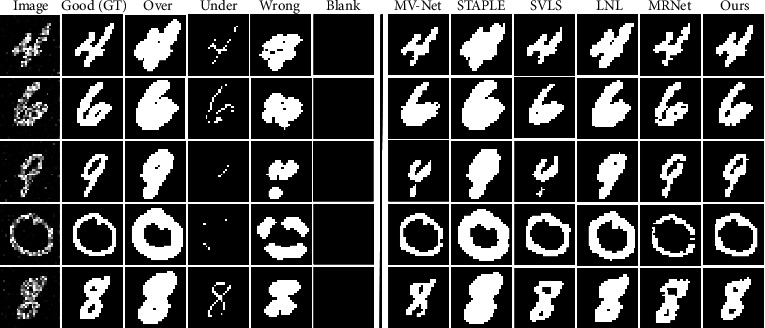
Visualization of five raters' annotations and predictions of six methods on the synthetic MNIST test set.

As shown in Table 2, our proposed method consistently achieves superior performance compared with other methods. For value *𝒟*, our method outperforms the SOTA method by 1.13% on the synthetic MNIST dataset.  Figure 5 shows the visualization results, wherein our method recovers the most realistic result from several annotations containing obvious human errors. Additionally, compared to the suboptimal MRNet method, the segmentation results predicted by the proposed method are smoother and more structured at the edge. For the real-world dataset RIGA, the performance improvement is especially prominent for the retinal OC segmentation, where the inter-rater variability is more significant, with a 2.29% increase in *𝒟* value over the current best method (listed in Table 2).

**Figure 6:**
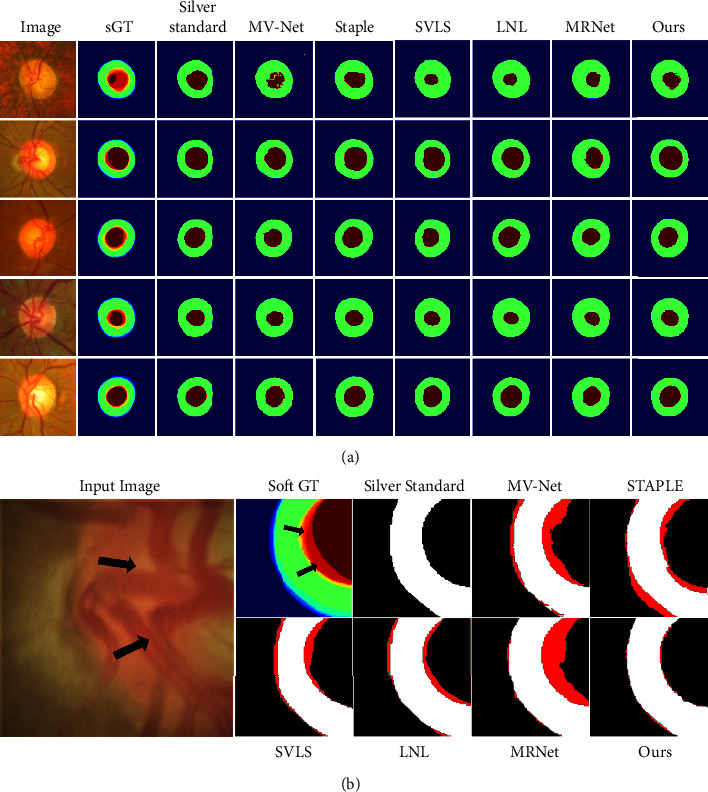
(a) Visualization of segmentation predictions on RIGA test set. (b) An example of a visualized partial map of predicted errors.

Figure 6(a) visualizes five examples of the silver standard and the corresponding segmentation results predicted by six different methods. As shown in Figure 6(b), the edge of OC occluded by blood vessels in the area with high inter-rater divergence (indicated by arrows) and, similarly, the high-incidence area of misprediction (red areas) by other methods. Compared to other methods, the proposed method shows lower prediction errors in the aforementioned area, demonstrating the robustness of our method to difficult features.

### 4.6. Ablation Studies

In this section, ablation studies are performed on the RIGA dataset over each component of the proposed method, including label smoothing (LS), consistency regularization (CR), and asymmetrical regularization (AR), as listed in Table 3. Meanwhile, the effect of different label smoothing techniques including GLS and Sharpen and SVLS on the performance of our method is also explored. The baseline model is the UNet trained using soft labels of average raters. All experiments are performed with the same network structure and training hyperparameters, Sections 4.2 and 4.4. *𝒟*_ave_ represents the average value of *𝒟*_*OC*_ and *𝒟*_*OD*_.

As shown in Table 3, the segmentation performance of the model reaches SOTA when all components are activated. As we sequentially remove the proposed components from the U-Net Baseline, the model performance degrades gradually. In particular, the inclusion of CR improved the baseline by 1.10%. Then, the combination of CR and AR yielded an additional 0.55% improvement. This means that, in areas with higher rater inconsistency in annotations, the potential representation of image features is more reliable than in uncertain annotations. It is proved experimentally that features are also one of the important causes of observer variability rather than just the rater knowledge. In addition, adding SVLS alone improves *𝒟*_ave_ of baseline by 0.52% while utilizing it with CR and AR jointly improves *𝒟*_ave_ of the proposed method without SVLS by 1.05%. It demonstrates that the positive effects of CR and AR are further strengthened under the threshold of uncertainty with SVLS.

### 4.7. Generalization Capability

**Figure 7:**
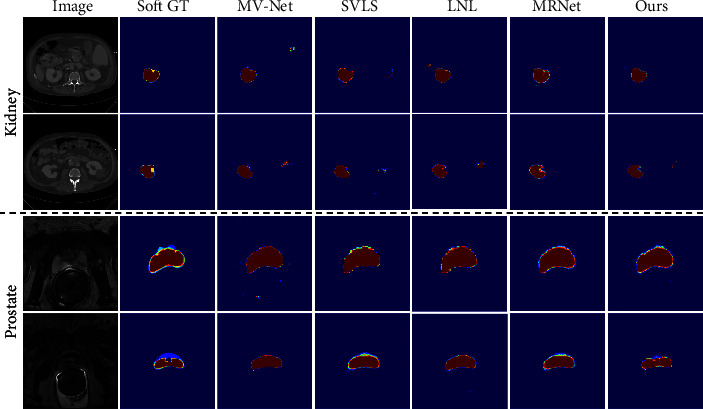
Visualization of segmentation predictions on the QUBIQ-kidney and prostate test sets.

To further verify the generalization capability of the proposed method, we additionally perform experiments on the kidney segmentation task of the QUBIQ multirater segmentation challenge. We use the same multithreshold scores *𝒟*^(soft)^ and *ℐ*^(soft)^ as QUBIQ challenge, which can better evaluate the ability of the model to reflect potential inter-rater agreement/disagreement. Specifically, after the GT and prediction are binarized at multiple threshold levels (0.1, 0.3, 0.5, 0.7, and 0.9), the *𝒟* and *ℐ* metrics averaged across five thresholds are *𝒟*^(soft)^ and *ℐ*^(soft)^. As listed in Table 4, compared to the comparative methods, the proposed method achieves optimal performance on the QUBIQ-Prostate dataset and achieves suboptimal performance on the QUBIQ-Kidney dataset. Furthermore, the advantage of a low number of parameters facilitates the application of our method to other multirater datasets. Several representative examples of the comparison methods for such two datasets are visualized in Figure 7.

## 5. Conclusion

In this article, we focus on the utilization of rich annotation information from multiple clinical raters, which are relatively less explored but widely presented in medical image segmentation. Based on the deep learning method using soft labels, we proposed a local self-ensembling learning model related to pixelwise variance with the intention of reducing the reliance upon uncertain local labels and optimizing the feature space. Our method achieves performance improvement over the soft labels' learning method without requiring the introduction of extra parameters and structures. In addition, we incorporate structural uncertainty into soft labels via the label smoothing technique to further improve segmentation performance level. Empirical experiments demonstrated the overall superior performance of our method on a synthetic dataset and a real-world dataset. Our method provides a solution for automatically learning a reliable clinical-aided diagnosis system using multirater annotations.

## Figures and Tables

**Table 1:** The segmentation performance (mean ± standard deviation) of different combinations of loss functions: supervised loss + consistency regularization loss.

Loss	*μ*	*λ*_2_	Performance
			*𝒟* ± std(%)
CE and CE	0.005	0.5	**94.09 ± 0.51**
CE and DL	0.005	0.5	93.55 ± 0.78
DL and DL	0.005	0.1	92.39 ± 1.09
DL and CE	0.005	0.1	91.15 ± 1.16
FL and FL	0.005	0.5	89.91 ± 0.59

**Table 2:** Quantitative results with different strategies on the MNIST and RIGA test set.

Methods		MNIST		RIGA			
		*𝒟*(%)	*ℐ*(%)	*𝒟*_*OD*_(%)	*𝒟*_*OC*_(%)	*ℐ*_*OD*_(%)	*ℐ*_*OC*_(%)
Hard	Mode-UNet	62.89	57.30	96.90	82.41	94.62	75.09
	MV-UNet	89.14	80.59	97.03	84.92	94.35	73.47
	STAPLE-UNet	82.26	74.51	96.28	85.37	92.84	75.68

Soft	Average-UNet	90.54	82.85	97.04	85.40	94.52	76.58
	GLS-UNet	87.32	78.29	96.14	86.83	93.71	75.95
	Sharpen^(*T*=0.5)^-UNet	90.50	81.03	96.85	84.71	94.33	77.18
	Sharpen^(*T*=1.5)^-UNet	87.67	80.13	96.77	86.13	93.82	77.90
	Mixup-UNet	86.61	78.58	96.83	84.72	94.02	75.18
	SVLS-UNet	90.32	82.05	97.40	86.09	94.95	76.87

SOTA	LNL	84.52	76.33	*97.67*	*87.56*	95.46	*78.76*
	MRNet	*93.63*	*88.09*	97.60	86.54	*95.78*	78.19

	**Ours**	**94.76**	**90.82**	**97.98**	**89.85**	**96.04**	**81.97**

The best results are highlighted, and the second best results are italic.

**Table 3:** Ablation experiment results on the RIGA dataset.

Module			Performance
LS	CR	AR	*𝒟*_*ave*_ ± std(%)
SVLS	√	√	**93.92** ± **0.56**
Sharpen^(1.5)^	√	√	93.55 ± 0.73
GLS	√	√	93.49 ± 0.63
SVLS	√		93.36 ± 0.71
Average	√	√	92.87 ± 0.65
Average	√		92.32 ± 0.82
SVLS			91.74 ± 0.78
Average			91.22 ± 1.06

**Table 4:** Quantitative results with different strategies on the QUBIQ-kidney and prostate test sets.

Methods	Kidney		Prostrate		#Parameters
	*𝒟* ^(soft)^(%)	*ℐ* ^(soft)^(%)	*𝒟* ^(soft)^(%)	*ℐ* ^(soft)^(%)	
MV-UNet	66.59	57.83	83.50	73.71	22.0M
STAPLE-UNet	65.01	56.31	83.36	73.69	22.0M
Average-UNet	69.33	58.21	85.82	77.02	22.0M
SVLS-UNet	70.04	58.65	86.11	77.38	22.0M
LNL	68.40	58.59	85.44	76.91	22.2M
MRNet	**71.36**	**60.43**	*87.39*	*78.14*	81.1M
**Ours**	*70.25*	*59.08*	**87.67**	**78.55**	22.0M

The best results are highlighted, and the second best results are italic.

## Data Availability

The data used to support the findings of this study are available from the corresponding author upon request.
